# Relationship Between Migraine and Abnormal EEG Findings in Children

**Published:** 2012

**Authors:** Habibe NEJAD BIGLARI, Alireza REZAYI, Hamid NEJAD BIGLARI, Mehdi ALIZADEH, Farzad Ahmadabadi

**Affiliations:** 1 Resident of Pediatrics, Mofid Children Hospital, Shahid Beheshti University of Medical Sciences, Tehran, Iran; 2 Fellowship of Pediatric Neurology, Assistant Professor of Pediatrics, Pediatric Neurology Research Center, Shahid Beheshti University of Medical Sciences, Tehran, Iran; 3 General Physician, Kerman University of Medical Sciences, Kerman, Iran

**Keywords:** Migraine, Children, Headache, Abnormal EEG, Epilepsy, Relationship

## Abstract

**Objective:**

Migraine is a disabling illness that causes absence from school and affects the quality of life. It has been stated that headache may represent an epileptic event. EEG abnormality is a prominent finding in children with migraine. The aim of this study was to evaluate EEG abnormalities in children with migraine.

**Materials & Methods:**

Two-hundred twenty-eight children were enrolled into the study. Evaluation and following of cases was performed by one physician, paraclinical tests were used to increase the accuracy. The study was conducted under the supervision of pediatric neurology masters and the selected cases were from different parts of the country.

**Results:**

Comparing EEG abnormalities in different types of migraine revealed that there is an association between them. There was also a significant difference between EEG abnormalities in different types of aura. Migraine type was associated with the patient’s age. Sleep disorders were more common in patients with a positive family history of seizure.

**Conclusion:**

Our study dosclosed migraine as a common problem in children with abnormalities present in approximately 20% of the patients. Migraine and abnormal EEG findings are significantly associated.

## Introduction

Approximately 5% of children have recurrent episodes of headache that are consistent with the diagnosis of migraine. Migraine without aura is the most common form of migraine in children constituting approximately 60% of the cases.

Seizure disorders although less frequent than migraine, are still very important in pediatrics due to the great impact on children health. The association between these two important diseases seem likely, but the pathophysiologic mechanisms are unclear (1). Similar to primary headache disorders, seizure disorders are chronic neurological pathologies with episodic manifestations (2). Migraine and epilepsy share the same risk factors (e.g. positive familial history), triggers (e.g. sleep deprivation) and prophylactic drugs (e.g. valproate sodium) to some extent, suggesting the possibility of associations between them. The correlation between the type of aura and the type of seizure has been detected in pediatric migraine that has also revealed the relationship between EEG abnormality and the type of aura and epileptic syndromes. In families of children with migraine, there is a higher incidence of migraine and epilepsy compared to the normal population.

Besides, sleep disorder is common in these children (3). The clear pathophysiology of the comorbidity of migraine and seizure disorders is unknown, but shared environmental and genetic factors are the basic concepts in both diseases (4). Suggested mechanisms include an increased cortical excitability accounting for the increased risk of migraine and epilepsy (5) and seizures may be triggered by migraine. This condition is called migralepsy by the International Classification of Headache Disorders (ICHDII) (6, 7). EEG that may be presumed as the mirror of brain activity was used as a tool in this study for disclosing the association between migraine and seizure disorders.

## Materials & Methods

The study population were children with the diagnosis of migraine who were referred to the neurology clinic of Mofid children’s hospital. The diagnosis of migraine was based on IHS diagnostic criteria (Table 1).

**Table 1:** Diagnostic Criteria for Migraine Without Aura

A. At least five headache attacks lasting 4-72 hours (untreated or unsuccessfully treated) which have at least two of the four following characteristics: 1. Unilateral location 2. Pulsating quality 3. Moderate or severe intensity (inhibits daily activities) 4. Aggravated by walking stairs or similar routine physical activity4. Aggravated by walking stairs or similar routine physical activity B. During headache at least one of the two following symptoms occur 1. Phonophobia and photophobi 2. Nausea and/or vomiting

Inclusion criteria for patients were an age of less than 12 years, diagnosis of migraine according to the mentioned criteria and accessible medical records. The exclusion criterion was presence of concomitant organic or structural disorders. A questionnaire including demographic data, familial history, triggering factors and imaging study results was used to collect data. All patients were examined by one physician and based on the severity of the headache a score from 1 to 3 was assigned to the patients. Score 1 was assigned for no impact on daily activities, score 2 mentioned partial impairment and 3 indicated impaired functioning. The sample size was 228, based on α=5%, d=6.5% and p=50% and n = z^2^p (1-p)/d^2^ equation.

Examination of the patients by one physician, using paraclinic tests and supervision of the study by masters of pediatric neurology, increased the internal and external validity of the study. Patients were free to leave the study whenever they wanted and consent was obtained to use their data. After collecting the data, and SPSS 19 for Windows (SPSS Inc., Chicago, Illinois, USA) was used for statistical analysis.

## Results

Two-hundred twenty-eight patients ( age range, 2-14 years) were enrolled into the study of which four (1.8%) were under 4 years old, 89 (39%) were 4-8, 127 (55.7%) were 8-12 and eight (3.5%) were above 12 years. Onehundred four (45.6%) of the cases were girls and 124 (54.4%) were boys. Totally, 165 (72.4%) of the cases had migraine without aura and 63 (27.9%) of them had migraine with aura. The frequency of visual aura was 71.4% and this figure was 28.6% for auditory aura. The duration of headache was less than 1 hour in 98 (43%) children, 1 to 2 hours in 86 (37.7%) and more than 2 hours in 44 (19.3%) of the children. The classic type of migraine was seen in 53 (23.4%) cases, common type in 144 (63.2%) cases, migraine variant in 19 (8.3%) cases and epileptic syndromes in 12 (5.3%) of the cases. Familial history was positive in 163 (71.5%) of the patients. Seizure history with or without fever was positive in 69 (30.3%) of the patients. Seizure type was simple FC in 32 (46.4%) cases, complex FC in seven (10.1%), CPS in two (2.9%), SPS in three (4.3%) cases, absence in two (2.9%) cases and GTC in 23 (33.3%) of the cases. Familial history of seizure was positive in 58 (25.4%) children. EEG recording was performed in one center and a pediatric neurologist reported the EEGS blindly. EEG findings were abnormal in 44 (19.3%) of the patients. All the patients had normal imaging results. Comparing EEG abnormalities in different types of migraine revealed that there was an association between them (P = 0.01). There was also a significant difference between EEG abnormalities in different types of aura (P = 0.03). There was no significant association between migraine types and different age groups, but age (not age group) can affect the type of migraine (P = 0.04). Sleep disorders were more common in patients with a positive family history of seizure. The most common EEG abnormalities in our study groups were epileptic and paroxysmal discharges including spike, sharp and slow and spike waves. Spike and sharp waves were seen in the occipital lobe in nine patients and temporal lobe discharges were seen in three patients with epileptic syndrome. Both patients with absence seizure had 3 Hz spike and slow waves and three patients with CPS seizure had poly spikewaves in the temporal lobe. Ten percent of the migraine patients without a history of seizure had abnormal paroxysmal discharges in their EEG.

**Fig 1:**
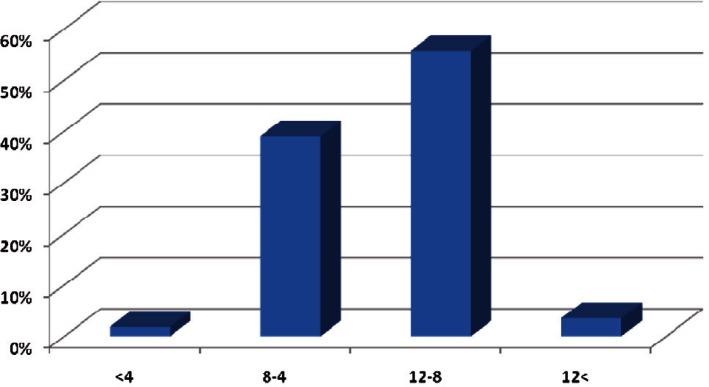
Frequency of different age groups that enrolled in our study

**Fig 2:**
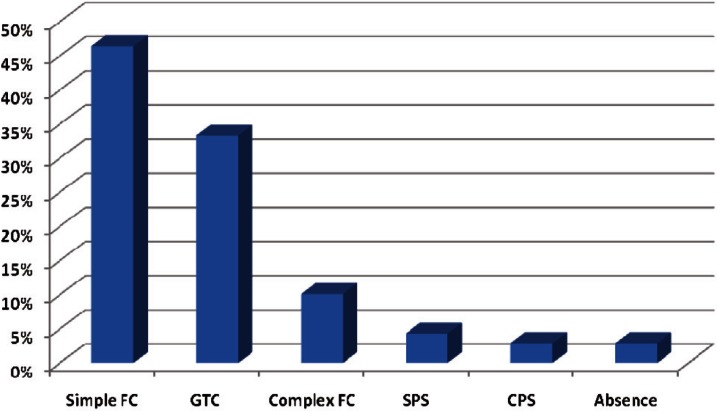
Frequency of different types of seizure inour study

**Fig 3:**
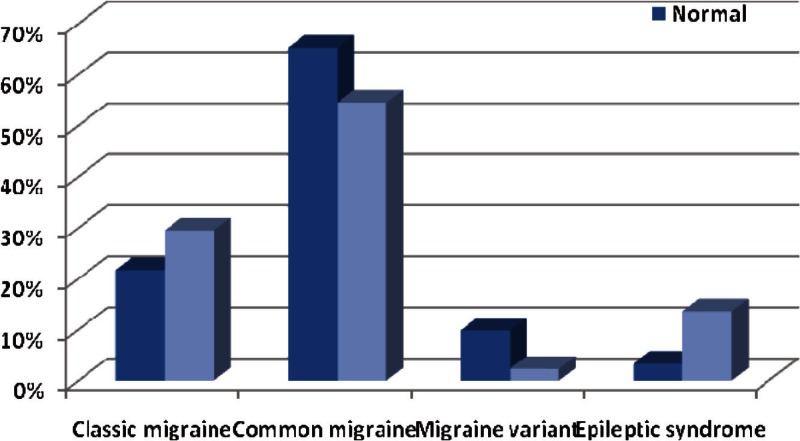
Comparing EEG results in different types of migraine in children

## Discussion

Migraine without aura is the most common type of migraine in children. In patients with aura, the most common aura is visual. Only 20% of children with the diagnosis of migraine have more than 2 hours of headache and the familial history of migraine is positive in about 70% of the patients EEG abnormalities in the child with migraine headaches were reported as 27%, in a study performed by Holguin and Fenichel (9), 20%, in a study conducted by Chu and Shinnar (10) and 23% in a study carried out by Friedman and Pampiglione (11). In Kramer and colleagues study, epileptiform EEG abnormalities were found in 11% in both migraine and tension headaches (12). In our study, the history of seizure was positive in approximately one third of the patients and febrile seizure and GTC were the common types of seizures in these patients. EEG findings were normal in roughly 20% of the patients and these findings were congruent with previous findings. A new finding that should be considered was that a strong relationship between EEG abnormalities and the type of migraine was detected. There was also the same relationship between EEG abnormalities and the type of aura. Abnormal EEG was more common in patients with a positive history of seizure. Another finding was that in our study there was an association between the patient’s age and the type of aura that was not mentioned in prior studies. Although there was no significant association between migraine types and different age groups, age can affect the type of migraine (P=0.04). This conflict is due to the age distribution of our study group, which was not normal.

Based on these findings, we must consider that headache may represent an epileptic event, an aura of a major motor seizure, or a postictal period of a nonevident seizure (13).
